# Akt-mediated anti-apoptotic effects of substance P in Anti-Fas-induced apoptosis of human tenocytes

**DOI:** 10.1111/jcmm.12059

**Published:** 2013-04-11

**Authors:** Ludvig J Backman, Patrik Danielson

**Affiliations:** a Department of Integrative Medical Biology, Anatomy, Umeå UniversityUmeå, Sweden; b Department of Surgical and Perioperative Sciences, Sports Medicine, Umeå UniversityUmeå, Sweden

**Keywords:** tendinopathy, tendinosis, Neurokinin-1 Receptor, cell death, tachykinin, caspase-3, PARP, tendon cells

## Abstract

Substance P (SP) and its receptor, the neurokinin-1 receptor (NK-1 R), are expressed by human tenocytes, and they are both up-regulated in cases of tendinosis, a condition associated with excessive apoptosis. It is known that SP can phosphorylate/activate the protein kinase Akt, which has anti-apoptotic effects. This mechanism has not been studied for tenocytes. The aims of this study were to investigate if Anti-Fas treatment is a good apoptosis model for human tenocytes *in vitro*, if SP protects from Anti-Fas-induced apoptosis, and by which mechanisms SP mediates an anti-apoptotic response. Anti-Fas treatment resulted in a time- and dose-dependent release of lactate dehydrogenase (LDH), *i.e*. induction of cell death, and SP dose-dependently reduced the Anti-Fas-induced cell death through a NK-1 R specific pathway. The same trend was seen for the TUNEL assay, *i.e*. SP reduced Anti-Fas-induced apoptosis *via* NK-1 R. In addition, it was shown that SP reduces Anti-Fas-induced decrease in cell viability as shown with crystal violet assay. Protein analysis using Western blot confirmed that Anti-Fas induces cleavage/activation of caspase-3 and cleavage of PARP; both of which were inhibited by SP *via* NK-1 R. Finally, SP treatment resulted in phosphorylation/activation of Akt as shown with Western blot, and it was confirmed that the anti-apoptotic effect of SP was, at least partly, induced through the Akt-dependent pathway. In conclusion, we show that SP reduces Anti-Fas-induced apoptosis in human tenocytes and that this anti-apoptotic effect of SP is mediated through NK-1 R and Akt-specific pathways.

## Introduction

Studies have shown that the neuropeptide SP, originally known for its role in the afferent sensory nervous system, mediates multiple efferent pathways, such as those involved in cell proliferation 1, 2 and apoptosis 3. SP stimulates the neurokinin-1 receptor (NK-1 R) in various cell types, including human tendon cells, *tenocytes* 1. Expression of SP and the NK-1 R has been observed in human tenocytes *in vivo* 4. This is particularly the case for the tenocytes of tendons afflicted by *tendinosis*, a condition of chronic tendon pain (*tendinopathy*) and tissue changes such as hypercellularity, angiogenesis and collagen disorganization 5. Thus, in tendinosis tendons, SP is up-regulated in the tenocytes 6, and also the NK-1 R has been shown to be expressed at higher levels in tenocytes of tendinosis tendons as compared with those in controls 4. Furthermore, SP-positive nerves are also increased in tendinosis 7, 8.

Apoptosis is a prominent microscopic feature observed in tendinosis tissues 9, but the role of SP and the NK-1 R in the regulation of apoptosis and cell survival in tenocytes is poorly understood. It is possible that SP contributes to either excessive apoptosis and/or cell survival. We have recently shown that SP increases cell viability of tenocytes *in vitro* and that this is partly explained by an increased proliferation rate 1. However, it cannot be excluded that the increased cell viability also is a result of inhibition of apoptosis. In fact, it has been shown that SP has an anti-apoptotic effect in various cell types 3, 10, 11, either *via* inhibition of apoptotic pathways and/or activation of cell survival pathways 3, 12.

Akt, a protein kinase also called protein kinase B and known to be phosphorylated into its active form after stimulation with SP 3, plays a critical role in controlling the balance of cell survival and apoptosis 13. Activated/phosphorylated Akt (P-Akt) promotes cell survival and inhibits apoptosis, by inactivating pro-apoptotic members of the Bcl-2 family (which otherwise cause cytochrome C leakage from the mitochondria), and also by regulating expression of caspases (decreased expression) and of anti-apoptotic Bcl-2 family members (increased expression) 13, 14.

**Fig. 1:**
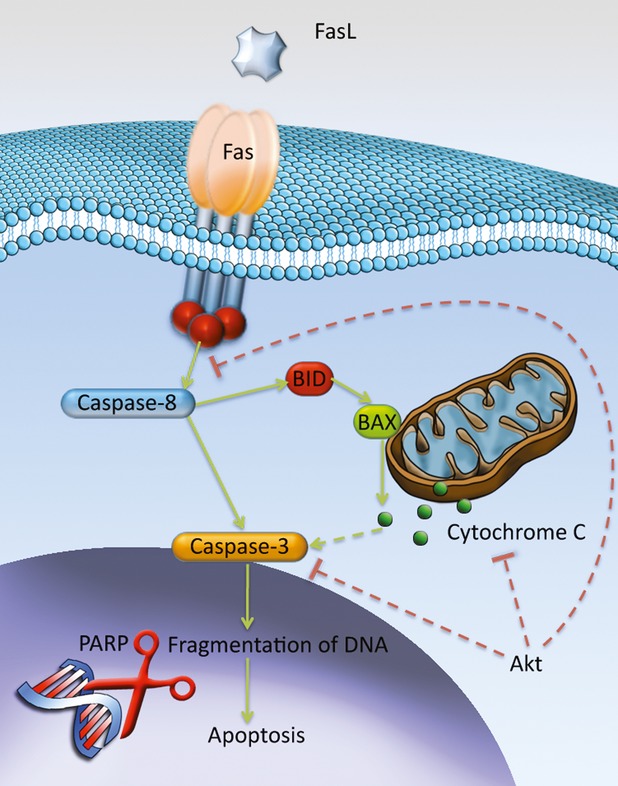
Mechanisms of FasL (Anti-Fas)-induced apoptosis and blocking by Akt. Binding of FasL to its receptor, Fas Receptor (Fas), results in activation of caspase-8, which subsequently can activate caspase-3 through two pathways; either *via* activation of pro-apoptotic Bcl-2 family proteins that cause cytochrome C leakage from the mitochondria, or through caspase-8 directly cleaving caspase-3 into activated (cleaved) caspase-3. One of the main targets for cleaved caspase-3 is cleavage of poly ADP ribosome polymerase (PARP), which is involved in the fragmentation of DNA, *i.e*. apoptosis. The protein kinase B, Akt, is known to prevent this apoptotic cascade indirectly through inhibiting activation of caspase-8, through inhibiting leakage of cytochrome C, and through inhibition of caspase-3 (illustrated with dotted line). Illustration by Gustav Andersson (copyright with artist).

Akt activation is known to protect cells against apoptosis agents belonging to the TNF family of death ligands, such as the Fas ligand (FasL) 15. Binding of FasL to its receptor (Fas or FasR) results in recruitment and activation of procaspase-8. Subsequently, caspase-8 can activate caspase-3 through two pathways; either through activation of pro-apoptotic Bcl-2 family proteins that cause cytochrome C leakage from the mitochondria, or through caspase-8 directly cleaving caspase-3 into activated/cleaved caspase-3 (c-caspase-3) 16. Ultimately, in the process of apoptosis, the DNA is fragmented after cleavage of poly ADP ribosome polymerase (c-PARP), which is one of the main targets of c-caspase-3 and established as an apoptotic response 3. See Figure 1 for an overview. It has been shown in preadipocytes that SP has an anti-apoptotic effect in FasL (Anti-Fas)-induced apoptosis, and that this effect of SP involves phosphorylation of Akt 17.

On the basis of all these previous studies, we hypothesize that SP mediates an anti-apoptotic response in tenocytes, thereby reducing the apoptosis seen in tendinosis, possibly by mechanisms involving the Akt pathway. Therefore, the aims of this study were to investigate (*i*) if Anti-Fas is a good apoptosis model for human tenocytes *in vitro*, (*ii*) if SP protects from Anti-Fas-induced apoptosis in tenocytes, and (*iii*) if an anti-apoptotic effect of SP is mediated through an Akt-dependent pathway. We have recently shown that human tenocytes in primary culture still express NK-1 R in passages used for experiments (making them susceptible to SP), and also that the cells continue to produce SP *in vitro* 1.

## Materials and methods

### Isolation of human Achilles tendon cells

Human Achilles tenocytes were isolated as previously described 1 and cultured in D-MEM supplemented with 10% foetal bovine serum (FBS; Invitrogen, Grand Island, NY, USA; 16,000), 1% pen-strep (code: 15140; Invitrogen) and 0.2% L-Glutamine (code: 25030; Invitrogen) at 37°C in a humidified atmosphere of 5% CO_2_ in air. At confluence, cells were harvested using trypsin 0.05% with EDTA (code: 25300; Invitrogen), re-suspended in medium and seeded at a 1:3 ratio. We have previously confirmed that these cells express scleraxis, tenomodulin and also collagen type I to a higher extent than collagen type III, which are all typical characteristics of tenocytes 1, 18.

In this study, a total of five different biopsies have been used from three males and two females with an age span of 21–59 years at the time of biopsy collection. All experiments were performed at least three times, and in at least two different patients, to confirm the results. No differences in behaviour or phenotype of cells from different patients were noticed during culturing, nor were any differences in response to SP observed.

The samples were collected from healthy donors, defined as voluntary individuals having no history of Achilles tendon pain and demonstrating no structural or vascular changes in colour Doppler ultrasound examination. They had all given informed consent. The study was approved by the Regional Ethical Review Board in Umeå and was performed according to the principles of the Declaration of Helsinki.

### Experimental condition

All cells for the experiments were serum-starved in 1% FBS 1 day before the start of the experiment. The condition of 1% FBS was used throughout the course of all experiments. The serum-starved cells appeared healthy and showed a steady increase in the metabolic activity over several days time.

### Substances for experimental design

In all experiments, SP (code: 05-23-0600; Calbiochem, San Diego, CA, USA) was used at concentrations of 10^−7^, 10^−8^ and/or 10^−9^ M, the NK-1 R antagonist, L-733,060 hydrochloride (code: 1145; Tocris, Minneapolis, MN, USA) was used at concentrations of 10^−6^, 10^−7^ and/or 10^−8^ M, FasL (Anti-Fas; code: 05-201; Millipore, Billerica, MA, USA) was used at a concentration of 250 ng/ml and/or 500 ng/ml, the Pan-Caspase inhibitor Z-VAD-FMK (code: G7231; Promega, Madison, WI, USA) was used at a concentration of 10 μM, and the Akt Inhibitor V, Triciribine (code: 124012; Calbiochem, Darmstadt, Germany) was used at a concentration of 40 μM. SP, the NK-1 R inhibitor, Z-VAD-FMK and the Akt inhibitor was incubated 30 min. before Anti-Fas.

### Haemotoxylin and eosin

Cells cultured on a 4-well chamber slide (code: 354104; BD Bioscience/BD Falcon, Bedford, MA, USA) at a concentration of 3.5 × 10^4^ cells/well were fixed for 5 min. in 20% paraformaldehyde in 0.1 M phosphate buffer (pH 7.4). After washing, cells were stained in haematoxylin solution (code: 1312002; Harris HTX Histolab; Gothenburg, Sweden) for 5 min. After a quick wash in distilled water, an incubation of 15 sec. in 0.1% acetic acid was performed. After 4 min. in warm water, the slides were incubated 5 min. in Eosin Y diluted in ethanol (code: E6003; Sigma-Aldrich, St louis, MO, USA). Before staining was performed in mounting medium, the slides were incubated in 95% EToH three times for 2 min. each.

### Immunocytochemistry

Cells cultured on an 8-well chamber slide (code: 354118; BD Falcon) at a density of 1.5 × 10^4^ cells/well were stained for c-PARP (rabbit polyclonal primary antibody; code: 9541; Cell signal, Danvers, MA, USA) and the Fas Receptor (mouse monoclonal primary antibody; code: 05-201; Millipore).

Cells were fixed in 2% paraformaldehyde in 0.1 M phosphate buffer (pH 7.4) for 5 min. followed by washes in PBS. Incubation with swine normal serum (SNS) at a concentration of 1:20 for 15 min. was performed before incubation with primary antibody against c-PARP at a concentration of 1:100 for 60 min. at 37°C. After additional washes, cells were again incubated with SNS to block unspecific binding for 15 min. and then incubated with a tetramethylrhodamine isothiocyanate (TRITC)-conjugated swine anti-rabbit secondary antibody (code: R0156; Dako, Copenhagen, Denmark) at 37°C for 30 min. at a concentration of 1:40. Before mounting in Vectashield Hard Set Medium with DAPI (code: H-1500; Vector Laboratories, Burlingame, CA, USA), cells were washed.

The same protocol for detection of Fas Receptor was used except that blocking was performed with 1% bovine serum albumin (BSA) in PBS, that the primary antibody incubation was carried out in room temperature (RT) for 120 min. at a concentration of 5 μg/ml, and that the secondary antibody was a fluorescein isothiocyanate (FITC)-conjugated goat antimouse IgM (code: F9259; Sigma-Aldrich) used at a concentration of 1:200 and incubated at RT for 90 min.

### TUNEL assay

Cells cultured on an 8-well chamber slide (code: 354118; BD Falcon) at a density of 1.5 × 10^4^ cells/well were stained for fragmented DNA using DeadEnd Fluorometric TUNEL system (code: G3250; Promega).

Cells were fixed in 2% paraformaldehyde in 0.1 M phosphate buffer (pH 7.4) and after washing, cells were permeabilized in 0.2% triton x-100 diluted in PBS for 5 min. and thereafter washed again. After equilibration for 10 min. at RT, cells were incubated with the nucleotide mix for 60 min. at 37°C. The reaction was thereafter stopped by adding the 2× saline-sodium citrate (SSC) solution and incubated for additional 15 min. After cells were washed and mounted in Vectashield Hard Set Medium with 4,6-diamidino-2-phenylindole (DAPI; code: H-1500; Vector Laboratories), they were subjectively analysed by fluorescence microscope, Zeiss Axioskop 2 Plus microscope with epifluorescence (Zeiss, Thornwood, NY, USA) and an Olympus DP70 digital camera (Olympus, Tokyo, Japan).

### Western blot

Cells were lysed in buffer [150 mM Sodium chloride, 1% Triton, 0.5% Sodium deoxycholate, 0.1% Sodium Dodecyl Sulphate (SDS), 50 mM Tris, pH 8.0], mixed with Laemmli sample buffer (code: 161-0737; Bio-Rad, Hercules, CA, USA) supplemented with 5% beta-mercaptoethanol (code: Me0095; Scharlau, Barcelona, Spain), boiled and separated by electrophoresis at 160 V for 45 min. in a 12% TGX gel. The proteins were then transferred to a polyvinylidene fluoride transfer membrane (PVDF membrane) at 100 V for 60 min. After the membranes were blocked with 5% dried milk in tris-buffered saline with tween 20 (TBS-T) for 60 min., they were incubated overnight at 4°C with primary antibody against c-PARP (code: 9541; Cell signal) at a concentration of 1:1000, c-caspase-3 (code: 9664; Cell signal) at a concentration of 1:1000, P-Akt (code: 9271; Cell signal) at a concentration of 1:2000 and Beta-actin (code: 4967; Cell Signal) at a concentration of 1:1000. As positive controls, Akt control cell extracts (code: 9273; Cell signal) and jurkat apoptosis cell lysates (code: 2043; Cell signal) were used for P-Akt and activated apoptotic cascades respectively. After membrane was washed in TBS-T, the secondary antibody, HRP-conjugated secondary antibody, 1:2000 (code: 7074; Cell Signal) was incubated for 60 min. at RT. After additional washing, bands were detected using chemiluminescent HRP substrate (code; RPN2132; GE Healthcare, Little Chalfont, Buckinghamshire, UK) and visualized on high-performance chemiluminescence film (code: 28-9068-38; GE healthcare).

Re-blotting of the membrane with β-actin was performed to confirm equal loading. This was performed after treatment with Western blot stripping buffer (code: 21059; Thermo Scientific, Rockford, IL, USA) at 37°C for 25 min. with constant agitation.

### Crystal violet

Cell viability was measured using crystal violet staining as previously described 1. Briefly, 1.5 × 10^5^ cells were seeded in a 6-well plate and fixed in 1% glutaraldehyde after being washed in PBS to remove any non-adherent cells. Cells were then stained in 1% crystal violet (code: C3886; Sigma-Aldrich) for 30 min., which was followed by thorough washing in water. Air-dried cells were then permeabilized in 30% methanol and 10% acetic acid before the absorbance was read at 590 nm in a 96-well plate. Experiments were performed in triplicate.

### Lactate dehydrogenase (LDH) assay

To prepare samples for the LDH assay (code: 11 644 793 001; Roche Applied Science, Mannheim, Germany), cells were seeded at a density of 7.5 × 10^4^ cells/well in 12-well plates and serum-starved overnight before being exposed to treatments. At the end of the experiment, supernatant was collected from each well and centrifuged at 400 *g* for 5 min. to remove any cells and cell debris. Afterwards, 100 μl was carefully removed without disturbing the cell pellet and stored at −80°C until all time-points were collected. To determine the LDH activity in the supernatant, 100 μl of freshly diluted reaction mixture, consisting of catalyst and dye solution, were mixed with 50 μl of supernatant and plated in a 96-well plate protected from light for 30 min. before absorbance was read. Absorbance was read at 490 nm. As a positive control, cells were lysed in 1% Triton X-100 (Kebo Lab, Stockholm, Sweden). Experiments were performed in triplicate.

### Statistics

Statistical calculations were done by the use of computer software (PASW Statistics 18.0.0; SPSS Inc., Chicago, IL, USA). One-way analysis of variance (anova), followed by the Bonferroni post-hoc test, was applied. Statistical significance was predetermined at *P* < 0.05.

## Results

### Phenotype of cells

As in previous studies on this model, the vast majority (ca. >95%) of cultured human tendon cells were, prior to the experiments, confirmed to be of tenocyte phenotype, as seen with the markers vimentin, scleraxis and tenomodulin, and also based on a high expression of collagen type I (see 18 for details on methods and results). This phenotype of the cells was preserved through the passages and serum concentrations used in the experiments. Very sporadically (ca. <1 in 20 cells), cells with extended dendritic-like processes were observed, indicating that a few nerve cells might be present (see 1).

### Presence of Fas Receptor (FasR), and cell morphology after Anti-Fas treatment

**Fig. 2:**
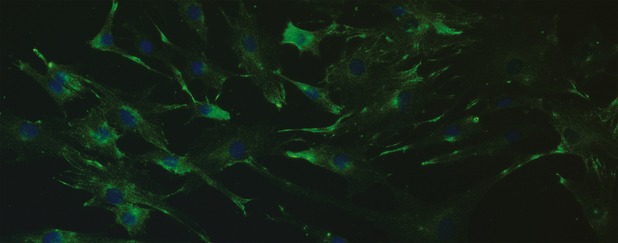
Immunocytochemical staining of primary human tendon cells shows immunoreactions for the Fas Receptor in almost all cells (green; FITC). Cells are counterstained with a nucleus marker (blue) using DAPI.

More or less, all primary tendon cells in culture were immunopositive for FasR, albeit to varying degrees, which provides a basis for apoptosis stimulation by FasL (Anti-Fas; Fig. 2).

Following 24 hrs of Anti-Fas treatment, about 25–30% of the cells had changed morphology, including cell shrinkage and nuclear fragmentation, which are cardinal signs of apoptosis. The apoptotic morphology was seen to a lesser extent when cells were simultaneously incubated with SP.

### Anti-Fas induces cell death

**Fig. 3:**
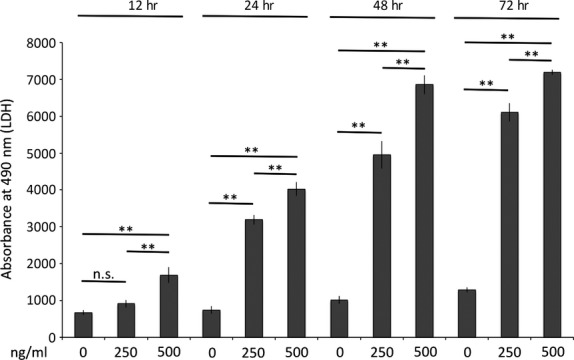
Anti-Fas treatment results in a time- and dose-dependent increase in cell death, as measured by the release of LDH, in cultures of primary human tenocytes. Concentrations of Anti-Fas used are indicated on the *X*-axis. There is a significant difference in effect between all concentrations at all time-points apart from the 12 hr exposure when comparing 250 ng/ml of Anti-Fas with the control for that time-point. Error bars show S.D. **P < 0.01.

Exposing cells to Anti-Fas resulted in time- and dose-dependent release of LDH, indicating a loss of membrane integrity, *i.e*. cell death (Fig. 3). Comparing 500 ng/ml with 250 ng/ml of Anti-Fas showed a significantly higher cell death at all time-points for the 500 ng/ml concentration. Nevertheless, 250 ng/ml of Anti-Fas was sufficient, at all time-points but 12 hrs, to cause a statistically significant increase in cell death as compared with control. Based on these results, 250 ng/ml of Anti-Fas was used for further experiments. The effect of Anti-Fas seemed to level out at 48 hrs (Fig. 3), still showing a clear dose–response effect at this time-point, and therefore this time-point was chosen for further experiments, evaluating end-point outcome in cell viability/cell death.

### SP dose-dependently reduces Anti-Fas-induced decrease in cell viability as well as dose-dependently reduces Anti-Fas-induced cell death

**Fig. 4:**
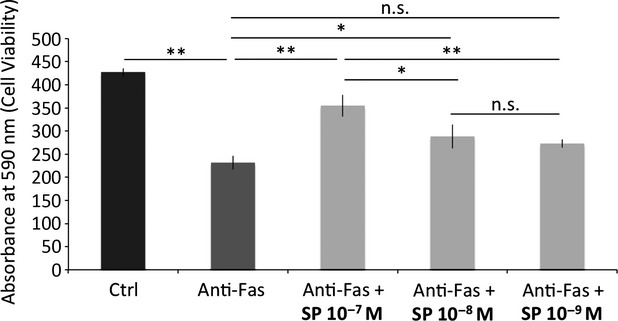
Anti-Fas treatment with 250 ng/ml for 48 hr results in a statistically significant reduction in cell viability in cultures of primary human tenocytes as compared with control. When cells are pre-treated with SP at concentrations of 10^−7^ M or 10^−8^ M, the Anti-Fas-induced decrease in cell viability is significantly reduced. This effect of pre-treatment with SP at a concentration of 10^−7^ M is significantly superior to the effect of both 10^−8^ M and 10^−9^ M SP. Pre-treatment with SP at a concentration of 10^−9^ M has no significant (n.s.) effect as compared with Anti-Fas alone. Error bars show S.D. *P < 0.05, **P < 0.01.

Anti-Fas treatment with 250 ng/ml for 48 hrs resulted in a statistically significant reduction in cell viability as compared with the control (Fig. 4). In comparison to exposure of Anti-Fas alone, pre-treatment with SP at a concentration of 10^−7^ or 10^−8^ M significantly reduced the Anti-Fas-induced decrease in cell viability (Fig. 4). Pre-treatment with SP at a concentration of 10^−9^ M had no statistically significant effect as compared with Anti-Fas alone. The effect of pre-treatment with SP 10^−7^ M resulted in significantly superior effect as compared with SP 10^−8^ or SP 10^−9^ M, showing a dose-dependent effect of SP.

**Fig. 5:**
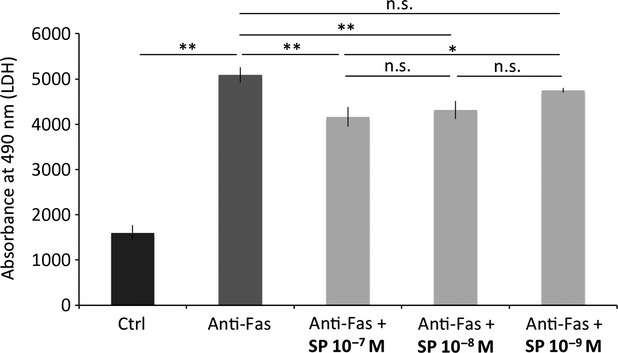
In comparison to the unstimulated control, exposure of primary human tenocytes to 250 ng/ml of Anti-Fas for 48 hr results in a significant increase in cell death (LDH-release). The Anti-Fas-induced cell death is significantly reduced when the cells are pre-treated with SP at concentrations of 10^−7^ M or 10^−8^ M, but this effect is not seen with 10^−9^ M of SP. There is a significant difference between cells pre-treated with 10^−7^ M SP as compared with 10^−9^ M SP, showing a dose-dependent response with the best protective effect of SP at a concentration of 10^−7^ M. Error bars show S.D. *P < 0.05, **P < 0.01.

Pre-treatment with SP at concentrations of 10^−7^ or 10^−8^ M significantly reduced the Anti-Fas- (250 ng/ml for 48 hrs) induced cell death, measured as LDH release, as compared with Anti-Fas alone (Fig. 5). This protective effect of SP was not statistically significant at a concentration of 10^−9^ M. Pre-treatment with SP at a concentration of 10^−7^ M resulted in a significantly higher reduction in cell death as compared with cells pre-treated with SP 10^−9^ M, showing a dose-dependent protective effect of SP with the best protective effect seen at a concentration of 10^−7^ M. Based on these results, SP at concentrations of 10^−7^ M was used for further experiments.

### SP effects on Anti-Fas-treated cells is mediated through a NK-1R specific pathway

**Fig. 6:**
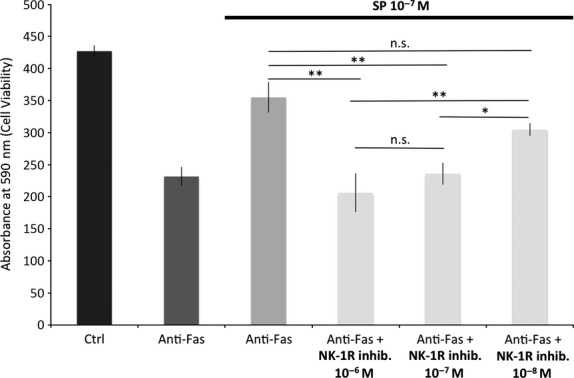
The increase in cell viability seen after pre-treatment with 10^−7^ M SP together with Anti-Fas, as compared to treatment with Anti-Fas alone (250 ng/ml; 48 hrs), is effectively blocked with the NK-1R inhibitor at concentrations of 10^−6^ M and 10^−7^ M, but not at a concentration of 10^−8^ M. The NK-1R inhibitor at a concentration of 10^−8^ M does not significantly (n.s.) inhibit the effect of SP. The blocking of SP's effect with the NK-1R inhibitor differs significantly with concentration; the NK-1R inhibitor at 10^−6^ M having a superior effect as compared with 10^−8^ M, and there is also a significant difference when comparing 10^−7^ M with 10^−8^ M. Error bars show S.D. *P < 0.05, **P < 0.01.

The increase in cell viability seen after pre-treatment with 10^−7^ M SP together with Anti-Fas, as compared to treatment with Anti-Fas alone (250 ng/ml; 48 hrs), was effectively blocked with the NK-1R inhibitor in concentrations of 10^−6^ and 10^−7^ M, but not at a concentration of 10^−8^ M (Fig. 6). A dose-dependent effect of the NK-1R inhibitor was confirmed (Fig. 6).

**Fig. 7:**
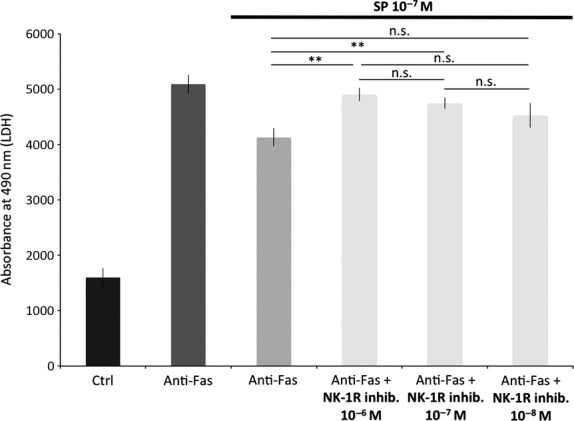
The effect of SP as a reducer of Anti-Fas-induced cell death is blocked when incubation with the NK-1R inhibitor at concentrations of 10^−6^ M or 10^−7^ M is added, but not when 10^−8^ M of the inhibitor is used. No significant difference (n.s.) between the different concentrations of the NK-1R inhibitor is, however, seen. Error bars show S.D. **P < 0.01.

The reduction in Anti-Fas-induced cell death that was seen after simultaneous incubation with SP, was not seen to the same extent when pre-incubation with the NK-1R inhibitor at concentrations of 10^−6^ and 10^−7^ M was performed (statistically significant; Fig. 7). However, the NK-1R inhibitor at the concentration 10^−8^ M did not have any similar significant effect (Fig. 7). Also, no significant difference between the different concentrations of the NK-1R inhibitor was seen.

### SP protects from Anti-Fas-induced apoptosis

**Fig. 8:**
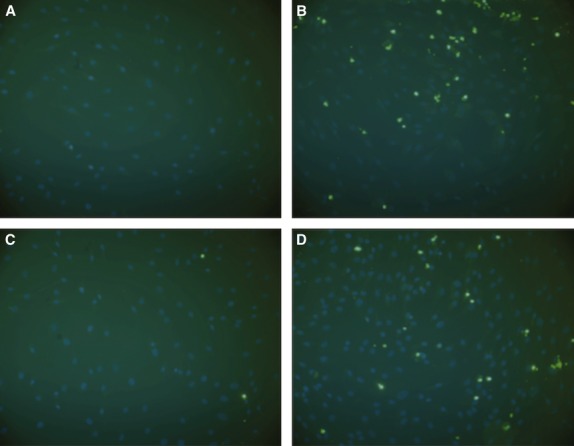
Fluorescent TUNEL staining (green; FITC) was performed to label fragmented DNA in primary human tendon cells to visualize apoptotic cells. Picture **A**, serving as control *i.e*. cells with no treatment, shows no reaction of TUNEL-positive cells, while picture **B**, containing cells treated with Anti-Fas (250 ng/ml) for 24 hrs, shows many positive, *i.e*. apoptotic, cells. This Anti-Fas effect is almost totally abolished when cells are pre-treated with 10^−7^ M SP as seen in picture **C**. In picture **D** cells are treated with Anti-Fas, SP, and the NK-1 R inhibitor (10^−6^ M), which results in many positive cells, showing that the anti-apoptotic effect of SP is mediated *via* NK-1 R. Nucleus staining is seen in blue using DAPI (A–D).

Neither cell viability nor release of LDH can distinguish between apoptosis and necrosis. However, fragmentation of DNA, detected with TUNEL assay, is specifically seen in cells undergoing apoptosis, and this phenomenon was microscopically seen after 24 hrs of Anti-Fas treatment in about 25–30% of the cells (Fig. 8B as compared with Fig. 8A). When cells were pre-treated with SP, less positive reactions were seen (Fig. 8C); this effect of SP being reduced when the NK-1 R inhibitor was also included (Fig. 8D).

**Fig. 9:**
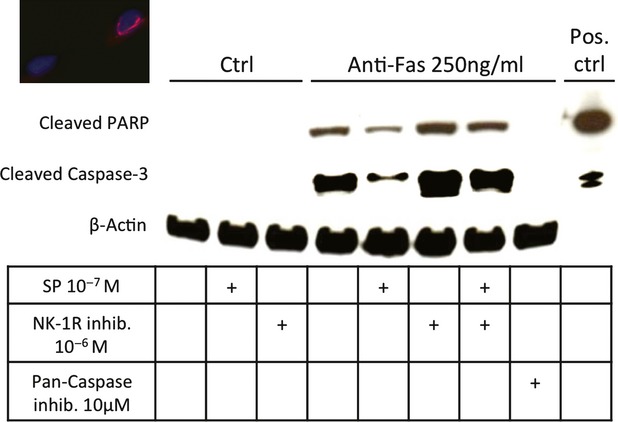
Western blot shows cleaved PARP and cleaved caspase-3 after Anti-Fas treatment for 12 hrs of primary human tendon cells in culture. No reactions at all are seen for the controls (Ctrl), *i.e*. when no Anti-Fas is added. The intensity of reactions for cleaved PARP and cleaved caspase-3 decrease when cells are pre-treated with SP. This effect of SP is blocked when the NK-1 R inhibitor is included. Incubation with the NK-1 R inhibitor and Anti-Fas alone results in slightly more intensive reaction than Anti-Fas alone. The Anti-Fas induced cleavage of caspase-3 and PARP is totally blocked when pre-treatment with a pan-caspase inhibitor (Z-VAD-FMK) is carried out. Positive controls (Pos. ctrl) are presented for cleaved PARP and cleaved caspase-3. Inset (upper left): Immunocytochemical staining using an antibody against cleaved PARP (red; TRITC) shows positive reactions after Anti-Fas treatment for 12 hrs. The nucleus is stained using DAPI (blue).

The active form of caspase-3 is the cleaved caspase (c-caspase-3), which in turn cleaves PARP (into c-PARP). On the level of protein, using Western blot, Anti-Fas (250 ng/ml) induced cleavage of caspase-3 and PARP in the tenocytes, after 12 hrs incubation (Fig. 9). This cleavage of both caspase-3 and PARP was decreased when cells were pre-treated with SP (10^−7^ M; Fig. 9). This effect of SP was blocked with the specific NK-1 R inhibitor at concentrations of 10^−6^ M. In fact, it was observed that the NK-1 R inhibitor alone with Anti-Fas treatment resulted in more cleavage of caspase-3 and PARP, as compared with Anti-Fas alone. The Anti-Fas-induced cleavage of caspase-3 and PARP was totally blocked when pre-treatment with the pan-caspase inhibitor Z-VAD-FMK (10 μM) was performed (Fig. 9). Neither SP nor NK-1 R alone resulted in any cleavage of caspase-3 or PARP (Fig. 9).

Using immunocytochemistry, positive reactions for c-PARP was seen in the cultured tendon cells after Anti-Fas treatment for 12 hrs (Fig. 9 inset), which was subjectively seen to a lesser extent in cases when SP pre-treatment was included.

### SP time-dependently increases Akt phosphorylation; an effect blocked with the NK-1R inhibitor

**Fig. 10:**
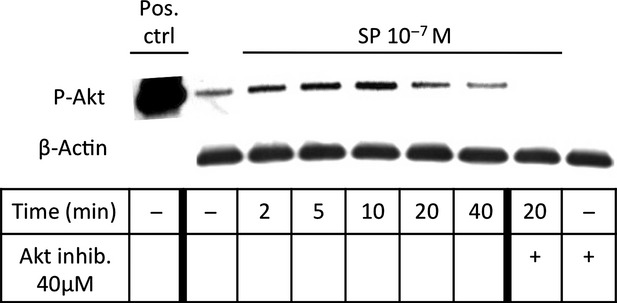
Western blot shows that SP increases phosphorylation, *i.e*. activation, of Akt (P-Akt) in human tendon cells in a time-dependent manner; peaking around 5–10 min. The Akt inhibitor abolishes all phosphorylation of Akt, regardless of SP being added or not. Positive control (Pos. ctrl) is presented for P-Akt. Time indicates the exposure time to SP in minutes.

Substance P time-dependently increased the phosphorylation of Akt. The peak effect was observed around 5–10 min. after exposure (Fig. 10). Phosphorylated Akt (P-Akt) was not seen after incubation with the Akt inhibitor, even when SP was added.

**Fig. 11:**
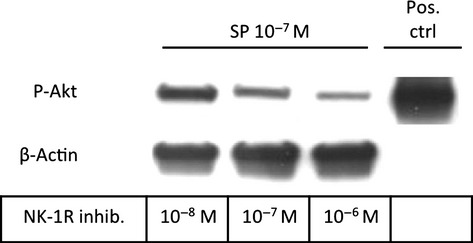
The NK-1R inhibitor reduces SP's phosphorylation (activation) of Akt (P-Akt) in a dose-dependent manner. Time-point: 10 min. after SP administration. The NK-1R inhibitor at a concentration of 10^−6^ M shows the best blocking effect of SP as compared to 10^−7^ M or 10^−8^ M. Positive control (Pos. ctrl) is presented for P-Akt.

The specific NK-1R inhibitor blocked the SP phosphorylation of Akt in a dose-dependent manner (Fig. 11). The blocking effect of the NK-1R inhibitor was the most efficient at a concentration of 10^−6^ M.

### The anti-apoptotic effects of SP is partly mediated by Akt

**Fig. 12:**
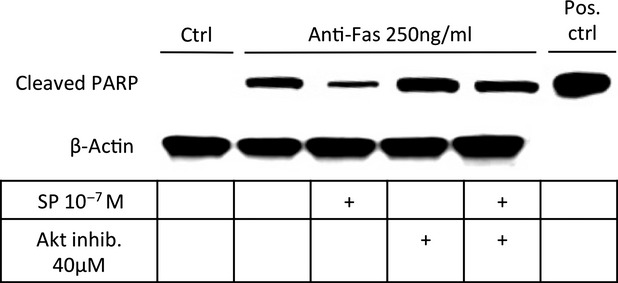
Western blot shows that Anti-Fas-induced cleavage of PARP is reduced by SP, and that this effect of SP is inhibited when an Akt inhibitor is added. No cleaved PARP is shown when no Anti-Fas is added in the control (Ctrl), whereas a clear band is seen for the positive control (Pos. ctrl).

The protective effect of SP on Anti-Fas-induced apoptosis, as measured by reduced cleavage of PARP (see Fig. 9), was blocked by the Akt inhibitor; further indicating that SP mediates its effect through Akt (Fig. 12).

### Anti-apoptotic effect of endogenously produced SP *via* Akt

**Fig. 13:**
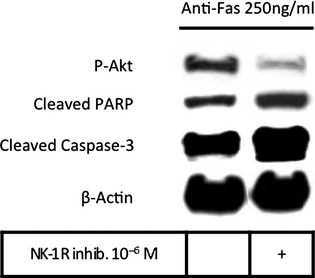
Anti-Fas treatment results in a cleavage of PARP and caspase-3, and this cleavage is increased when cells are pre-treated with the NK-1R inhibitor. The NK-1R inhibitor furthermore clearly reduces the endogenous phosphorylation (activation) of Akt (P-Akt). This seems to suggest an autocrine effect of endogenously produced SP in the human tendon cells.

When comparing the Anti-Fas-incubated tendon cells with cells incubated with Anti-Fas and the NK-1 R inhibitor together, there was a tendency towards increased c-caspase-3 (and also c-PARP) when the NK-1 R inhibitor was added (see Fig. 9 and also Fig. 13), suggesting that endogenously produced SP by the tendon cells 1 is also protective against Anti-Fas-induced apoptosis. Western blot for P-Akt on the same cells, showed that P-Akt was reduced after NK-1 R inhibition (Fig. 13), giving further evidence that endogenous SP might exert anti-apoptotic effects, *via* Akt, in Anti-Fas-induced apoptosis. See the last section of the Discussion.

## Discussion

In this study, we show that cultured human tenocytes undergo apoptosis following Anti-Fas treatment. Our results further show that SP, *via* NK-1 R, reduces Anti-Fas-induced apoptosis in a dose- and time-dependent manner in these cells, and that the anti-apoptotic effect of SP is mediated through an Akt-dependent pathway. Moreover, the results of the study seem to support the hypothesis that endogenous SP produced by tenocytes 1, exerts autocrine anti-apoptotic effects *via* NK-1 R.

### Methodological aspects

The methods of culturing primary human tendon cells used in this study have successfully been used in previous studies 1, 18. We have confirmed that the isolated cultured human tendon cells used in our study grow easily in culture and form networks with cell-cell connections (connexin 43; see 18), and that they are of tenocyte phenotype in the passages used for experiments, based on the expression of tenomodulin and scleraxis, both specific markers for tenocytes 19, 20. The cells were also shown to express a high level of collagen type I 18, which further confirms tenocyte characteristics 21. Of further interest for the results of this study, we have previously shown that these cells produce SP and express NK-1 R in passages used for these experiments 1.

In the present study, apoptosis was a vital end-point measurement for the hypothesis and aims. The release of LDH, which occurs when cells lose their membrane integrity, cannot distinguish between apoptosis and necrosis, nor can crystal violet assay, which is an indirect measurement of the number of cells. To confirm apoptotic events, we have therefore used TUNEL assay, which is a method designed to specifically detect cells undergoing apoptosis, as well as analysis of apoptotic events such as cleavage (*i.e*. activation) of caspase-3 and cleavage of PARP.

The induction of cell death by Anti-Fas, as measured with LDH, showed a clear dose- and time-dependent response. However, the dose–response shown for the effects of SP and the NK-1 R inhibitor was not as clear-cut, but a trend was seen with the most effective concentration for both SP and the NK-1 R inhibitor in accordance with the concentrations used by others 3, 17.

### Anti-Fas as a model for apoptosis in cultured tenocytes

We observed that human tenocytes abundantly express the Fas Receptor (FasR), making them susceptible to stimulation with FasL (Anti-Fas treatment). Indeed, we found that exogenously added Anti-Fas induced a dose- and time-dependent cell death (increase in LDH release). Anti-Fas concurrently reduced tenocyte cell viability, and furthermore induced apoptosis-specific events such as fragmentation of DNA and cleavage of caspase-3 and PARP, confirming an apoptotic effect of Anti-Fas.

It is known that inflammatory cells produce FasL 22, and that in cases of tendinosis, the degree of inflammatory cell infiltrates in the paratenon, the tendon sheath, is significantly increased 7, 23, 24. This makes it probable, that apoptosis of tendinosis *in vivo* 9 is partly explained by a FasL production by the inflammatory cells in the paratenon, which in turn stimulate the tenocytes expressing FasR to undergo apoptosis.

In our analysis of apoptotic events, cleaved caspase-3 and PARP are used. We show that Anti-Fas treatment cleaves/activates caspase-3 as well as cleaves PARP. It is well known that the main effect of c-caspase-3 is cleavage of PARP. However, the existence of c-PARP does not necessarily indicate apoptosis, as it is known to be able to occur as an independent event that can be disassociated from apoptosis 25. Furthermore, our study does not determine if caspase-8 directly activates caspase-3, and thereby indirectly cleaves PARP, nor if the cleavage of caspase-3 is a result of mitochondrial involvement and release of cytochrome C. Nevertheless, we show, by using the pan-caspase inhibitor (ZVAD-FMK), that the cleavage of PARP is caspase-dependent, as the pan-caspase inhibitor prevents the Anti-Fas-induced increase in cleaved PARP, and therefore we also demonstrate that the cleavage of PARP is apoptosis-dependent.

To summarize, in human tenocytes, Anti-Fas exposure is here confirmed to be a good model to induce apoptosis, at least when studying late apoptotic markers such as caspase-3 and PARP. Similar responses have also been observed in other cell types, such as epithelial cells 15, showing that Anti-Fas is a good apoptosis inducer, and human colonocytes 3, showing that apoptotic end-markers (caspase-3/PARP) are good to evaluate an apoptotic response in cultured cells.

### SP reduces Anti-Fas induced apoptosis in tenocytes

In this study, we show that SP dose-dependently protects from the Anti-Fas-induced decrease in cell viability and increase in LDH release (*i.e*. cell death), and also that it reduces the cleavage/activity of late apoptotic markers such as caspase-3 and PARP. However, when interpreting crystal violet results, one should bear in mind that SP has also been shown to have a proliferative effect in tenocytes 1, as well as in other cell types (such as preadipocytes 17 and human skin fibroblasts 10), and that this will affect the viability results. However, the LDH and crystal violet assays in combination with the specific apoptosis analyses, make it likely that the outcome from the LDH and crystal violet assays are a result of apoptosis. Nevertheless, the fact that SP in this study seems to have a less marked blocking effect on Anti-Fas-induced viability decrease, as compared with Anti-Fas-induced LDH increase, is logical and could be explained by SP's proliferative effect in these cells.

In this study, SP's preferred receptor, NK-1 R, was blocked with a NK-1 R inhibitor to confirm that the effect of SP is mediated *via* a NK-1 R specific pathway. Indeed, the effect of SP was reduced in a dose-dependent manner when cells were pre-treated with the NK-1 R inhibitor in different concentrations, confirming that SP mediates its effect *via* NK-1 R in human tenocytes. However, hypothetically, SP produced by the tenocytes 1, 4 can also bind to, and exert effects *via*, NK-2 R and NK-3 R; it is just a question of receptor availability and the concentration of SP 26. These two receptor subtypes were not investigated in this study, as NK-1 R is the dominant/preferred form 26, 27 and is also clearly expressed on tenocytes in human Achilles tendons 4, and as the results of the experiments here were clear-cut regarding the inhibition of SP effects when blocking the NK-1 R. Nevertheless, the fact that SP also has the possibility of binding to NK-2 R and NK-3 R, might explain why the use of the NK-1 R inhibitor did not completely abolish the effect of SP at all time.

The results that SP promotes decreased apoptosis, and also that it increases proliferation 1, in human tenocytes in culture, are effects corresponding to SP effects shown for other cell types as well. Thus, this double-edged response of SP has also been demonstrated in human colonocytes 2, 3, skin fibroblasts 10 and preadipocytes 17, in which SP stimulates proliferation and has an anti-apoptotic effect simultaneously.

### The anti-apoptotic effects of SP is mediated *via* activation of Akt

We show that in human tenocytes SP time-dependently activates Akt, a protein kinase that is well known to facilitate cell survival and to prevent apoptotic cell death 13. This is in accordance with previous results in other cell types 3, 28 in which SP also activates Akt. Furthermore, the reduction in Anti-Fas-induced apoptosis of human tenocytes seen after SP treatment in this study (as evident by decreased cleavage of PARP), is effectively blocked with an Akt inhibitor, showing that SP's effect is mediated, at least in part, *via* Akt.

Following exposure to SP, a plateau of phosphorylated Akt was seen after ca. 5–10 min. As phosphorylation of Akt is in the early cascade of an anti-apoptotic effect, this prompt Akt response after SP exposure is trustworthy, and, as it should, it precedes later responses, such as decreased cell death/apoptosis and increased viability. The prompt Akt response to SP is furthermore in accordance with a study on colonocytes 3, which in addition also had the same time-response for the SP induced reduction in end-point apoptosis markers, such as PARP cleavage.

In this study, we also confirmed that the phosphorylation of Akt was dose-dependently reduced when cells were pre-treated with the NK-1 R inhibitor. This confirms that the effect is specifically mediated *via* a SP-NK-1 R specific pathway.

There are other pathways, than the Akt pathway, that might mediate an anti-apoptotic effect in response to SP stimulation, which are not examined in this study. For instance, SP is known to induce an activation of NF-kappa B 29 which in turn can up-regulate the caspase-8 inhibitor FLIP, resulting in increased resistance to Anti-Fas-induced cell death 30.

### Apoptosis in tendinosis and the role of SP produced by tenocytes; concluding remarks

Tendinosis is known to be associated with a higher expression of apoptotic cells than normal tendon tissue 9, 31. The role of apoptosis in tendinosis is, however, unknown. A cardinal feature of tendinosis is furthermore the marked hypercellularity seen in the tendon tissue 5. The pathophysiology of such hypercellularity, as well as its possible function, is not yet clarified. However, in an animal model, we have shown that overload of the Achilles tendon induces elevated intratendinous SP production 32 as well as tenocyte hypercellularity 33. Most interestingly, the elevation of endogenous SP production was found to precede the increase in cell number, and exogenously administered SP further accelerated the hypercellularity 34. The fact that tenocytes respond to mechanical stress with increased SP production has furthermore been confirmed in human tendon cells *in vitro* 1.

It is possible, that the hypercellularity seen in tendinosis is an inappropriate response to mechanical stress, leading to defective tendon metabolism, and that the increase in apoptosis is an attempt for the tendon to maintain an environment with a balanced number of tenocytes, thereby limiting a process of excessive and possibly dysfunctional collagen synthesis (see for instance 35). If so, SP may, speculatively, have a dual detrimental role in the pathophysiology of tendinosis; harbouring the capacity of both stimulating proliferation of tenocytes 1 and inhibiting apoptosis of these cells, the latter shown by this study.

Our hypothesis is based on the assumption that there is an autocrine SP loop in human tenocytes, *i.e*. that the tenocytes produce SP, in response to mechanical stimuli, and this SP in turn affects the tenocytes themselves by stimulating NK-1 R on the cell surface. Several results seem to corroborate this hypothesis. Human tenocytes have been shown to produce SP *in vitro* (ranging in the concentration of 170 pg/2 × 10^6^ tendon cells) 1, and also to express the NK-1R both *in vitro* 1 and *in vivo* 4. In this study, the phosphorylation of Akt that is seen after Anti-Fas treatment (without exogenous SP being added) is reduced when the tenocytes are pre-treated with a NK-1R inhibitor (Fig. 13). As we also show, in this study, that SP effectively phosphorylate Akt in tenocytes, it is not far-fetched to speculate that endogenously produced SP, in an autocrine manner, binds to the NK-1Rs present on the cells, thus resulting in phosphorylation of Akt, a loop that is interfered with when the NK-1R inhibitor is added. The NK-1 R inhibitor together with Anti-Fas furthermore resulted in a higher expression of cleaved caspase-3 and PARP (Fig. 13), than did Anti-Fas alone, possibly suggesting that a protective, anti-apoptotic effect of endogenous SP is constantly present and here blocked by the NK-1 R inhibitor. Nevertheless, the present study, in conjunction with previous studies here cited, only provide indications of an autocrine SP loop in tenocytes. Future studies, using for instance siRNA technique to silence the SP expression, will have to elucidate this further.

To summarize, the two here mentioned effects of the NK-1 R inhibitor (reduced P-Akt and increased c-caspase-3/c-PARP) can be explained by two alternative sequences: (1) Endogenous SP is blocked, leading to decrease in P-Akt which in turn leads to increased apoptosis (Akt being a known anti-apoptotic protein kinase 13), or (2) endogenous SP is blocked, leading to increase in apoptosis (through alternative pathways than the Akt-dependent 29, 30), which in turn leads to a caspase-dependent cleavage/inactivation of Akt 15.

Finally, it should here be mentioned that as for the *in vivo* situation, alternative sources of ligands stimulating the NK-1 Rs of the tenocytes might, in addition to the tenocytes themselves producing SP, be SP-positive nerves in or around the tendon 8, 36, or speculatively cells of the tendon producing other tachykinins. It is thus known, that the tachykinin neuorokinin A (NKA) can also bind to NK-1 R with high enough affinity to elicit a biological response 37. Considering the fact that NKA is shown to be present in nerve endings of the paratenon of rat Achilles tendons 38 it is possible that NKA is another substance that might bind to the NK-1 R expressed by human tenocytes *in vivo*.

In conclusion, considering both its proliferative and anti-apoptotic effects, the results of this and previous studies identify SP as a potent regulator of the marked hypercellularity seen in tendon tissue as part of the pathology of tendinosis.
